# Genetic dissection of behavioral traits related to successful training of drug detection dogs

**DOI:** 10.1038/s41598-023-33638-6

**Published:** 2023-05-05

**Authors:** Yuki Matsumoto, Akitsugu Konno, Genki Ishihara, Miho Inoue-Murayama

**Affiliations:** 1 Anicom Specialty Medical Institute Inc., Yokohama, Kanagawa 231-0033 Japan; 2 Department of Animal Sciences, Teikyo University of Science, Uenohara, Yamanashi 409-0193 Japan; 3 Wildlife Research Center, Kyoto University, Kyoto, Kyoto 606–8203 Japan

**Keywords:** Genetics, Zoology

## Abstract

Drug detection dogs play integral roles in society. However, the interplay between their behaviors and genetic characteristics underlying their performance remains uninvestigated. Herein, more than 120,000 genetic variants were evaluated in 326 German Shepherd or Labrador Retriever dogs to profile the genetic traits associated with various behavioral traits related to the successful training of drug detection dogs. Behavioral breed differences were observed in ‘friendliness to humans’ and ‘tolerance to dogs.’ A genome-wide association study within both breeds identified 11 regions potentially associated with drug detection dog characteristics as well as ‘interest in the target’ and ‘friendliness to humans,’ which are related to drug detection abilities. Among them, 63 protein-coding genes, including *Atat1* and *Pfn2* known to be associated with anxiety-related or exploration behavior in mice, respectively, were located surrounding the identified candidate polymorphisms. This study highlights genetic characteristics associated with behavioral traits that are important for the successful training of drug detection dogs. Thus, these findings may facilitate improved breeding and training of these dogs.

## Introduction

Dogs are one of the oldest domesticated animals worldwide and play important roles in society, such as companion and working dogs. To ensure their suitability for such roles, artificial selection of behavioral traits and favoring of some genetic alterations has been performed to achieve specific breading goals. Indeed, collection of multiple evolutionary bottlenecks have led to the modern purebred dog breeds several hundred years ago^1,2^.

In particular, the modern working dogs, commonly known as detection or search dogs, can be trained to find objects with targeted odors (*e.g.*, drugs, explosives, missing humans, animals, and human diseases) by using their exquisite olfaction sensitivity and communicative ability with humans. Particularly, drug detection dogs find objects with drug odors and are used for interdicting the smuggling of prohibited drugs in transport (*e.g.*, airports or ports). In Japan customs, detection dogs are trained by positive reinforcement with the use of social rewards (*e.g.*, play or affiliative behavior), which is based on collaborative behavioral interaction between the dogs and their human handlers. However, only 30–50% of these animals are successfully trained to become effective drug detection dogs^3–5^, which suggests a high variability of the responsiveness to training of the dog candidates.

Over the past decades, the genetic basis of behavioral or personality traits (*i.e.*, consistent genetic patterns associated with individual behavior differences) in dogs has been widely assessed using several genetic markers previously associated with human behavior (*i.e.*, candidate gene approach). For example, the dopamine receptor D4 (*DRD4*) and serotonin transporter 1A (*5HTT*) genes were reported to be associated with activity-impulsivity, distractibility, and human-directed gazing behavior in dogs^5–8^. Previous studies, which addressed a wide range of study purposes and methodologies, have examined different genetic regions, populations of dogs, and behavioral traits. Nonetheless, additional behavioral genetic studies in dogs are still required to explore novel genetic regions based on the most recent accumulated research data.

A more comprehensive analysis using genome-wide single nucleotide polymorphisms (SNPs) was performed to uncover genes or genetic regions potentially associated with behavioral traits related to canine stereotypes^9^. In addition, genome-wide association studies (GWAS) covering a large number of genetic variants in cohorts with large sample sizes (n > 500) have successfully uncovered associations between various genetic variants and individual differences in behavioral traits (*i.e.*, personality) in dogs^10–13^. However, two GWAS using the Canine Behavior Assessment and Research Questionnaire (C-BARQ) obtained contrasting results, with one study failing to find significant gene-behavior associations^12^, whereas another study reported two genetic regions that were significantly associated with a subscale of the attachment/attention-seeking in C-BARQ^13^. However, the molecular function and pathway underlying the gene-behavior relationship remain unknown. For odor detection dogs, genetic polymorphisms in olfactory receptor genes, serotonin transporter genes, and oxytocin receptor genes have been found to be associated with performance in odor detection tasks and training success^4,5,14^. These previous reports suggest that the training success of a detection dog is genetically influenced by multiple genetic regions, whereas the effect of a single gene polymorphism may be weak. Additionally, studies of working dogs have suggested that differences in the responsiveness of a dog to the training are mainly due to their behavioral traits rather than sensory or morphological characteristics^4,15–18^. To expand our knowledge on behavioral genetics in working dogs, further research with a wider range of genetic analyses is needed. It should be noted that to date, to the best of our knowledge, no genetic markers, found by genome-wide analysis like GWAS, associated with odor detection ability and/or suitability for odor detection training are available. Information about such genetic markers may enable efficient training via genetic selection of dogs.

The present study aimed to evaluate the genetic characteristics associated with behavioral traits related to successful drug detection dog training in Japan based on genome-wide SNP genotyping and GWAS. We believe that this study can provide valuable genetic information, including candidate SNPs and genes that may be used in the future to identify dogs that will be more prone to respond successfully to drug training protocols.

## Results

### Breed and sex differences with respect to seven behavioral traits

**Figure 1:**
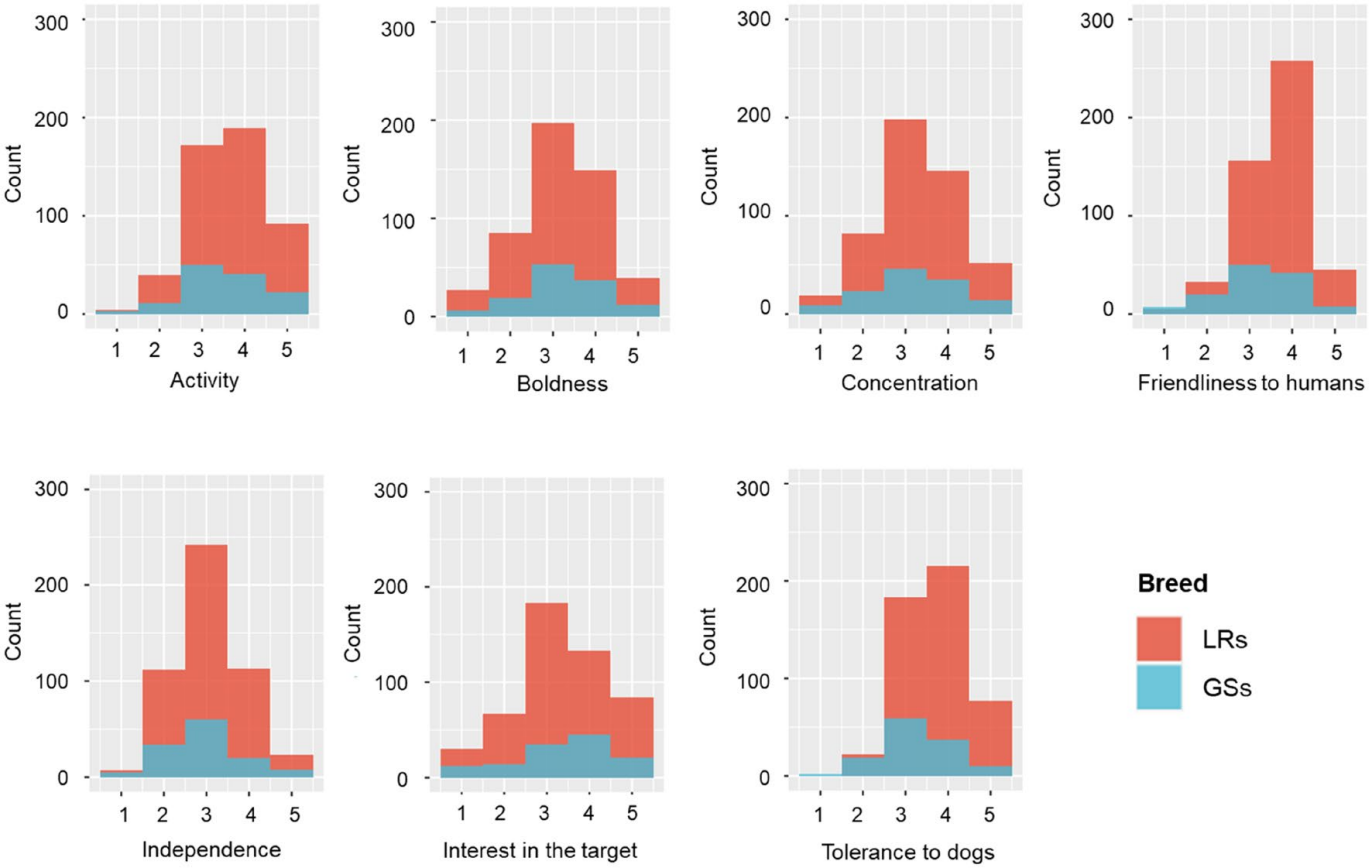
Histogram of the seven behavioral traits related to qualification (success of training) of drug detection dogs.

Differences in seven behavioral traits (*i.e.*, activity, boldness, concentration, friendliness to humans, independence, interest in the target, and tolerance to dogs) depending on the breed (German Shepherds [GSs] vs. Labrador Retrievers [LRs]), sex (male vs. female), and qualification status (qualified vs. unqualified) were evaluated using a rating scale (Fig. 1, Tables 1 and 2). LRs had higher scores for ‘friendliness to humans’ and ‘tolerance to dogs’ compared with GSs (Fig. 1). No significant effect of sex was observed in either dog breed. In GSs, we found that the qualified group significantly differed from the unqualified group in four behavioral traits (activity, boldness, ﻿concentration, and interest in the target). In LRs, all traits, except friendliness to humans, differed between the two qualification groups.

**Table 1:** Brief discription of seven behavioral traits.

Items	Description	Examples of dog behaviors
Activity	Amount of activity typically observed	The dog moves around and runs a lot; Dog moves quickly and is hyperactive
Boldness	Low fearfulness displayed toward unfamiliar environments	The dog does not show fear signals (e.g. ears down or a tail curled between legs) in novel situations
Concentration	Attention span during training	The dog keeps sniffing in a search trial; The dog is not distracted by irrelevant stimuli in a training session
Friendliness to humans	Willingness to interact with humans	The dog approaches humans in a friendly and playful manner; The dog does not show fear signals toward strangers
Independence	Self-confidence or willingness to work without having to rely on humans	The dog spontaneously engages in a search task without human involvement; The dog does not need the handler's help
Interest in the target	Degree of interest in the rolled towel (i.e., reinforcer)	The dog gets excited by rolled towels; when the dog bites a rolled towel, it is not easily removed by humans
Tolerance to dogs	Low aggressiveness towards other dogs	The dog approaches other dogs in a friendly manner; The dog does not show aggressive signals (e.g., growl, bark) toward dogs

* Score 1 (lowest) to 5 (highest) for all traits.

**Table 2:** Statistical test (*P* values) for expression of behavioral traits by sex, qualification and breed.

Items	GSs		LRs		Breed GS n = 127; LR n = 497
	Sex male n = 68; female n = 59	Qualification qu. n = 66; unq. n = 61	Sex male n = 288; female n = 209	Qualification qu. n = 165; unq. n = 332	
Activity	0.426	6.630 E−05*	0.442	< 2.200 E−16*	0.213
Boldness	0.332	8.050 E−06*	0.075	1.350 E−12*	0.594
Concentration	0.218	4.420 E−12*	0.084	< 2.200 E−16*	0.459
Friendliness to humans	0.666	0.022	0.242	2.660 E−06*	2.02 E−06*
Independence	0.821	0.760	0.005	0.001*	0.100
Interest in the target	0.185	8.930 E−13*	0.007	< 2.200 E−16*	0.472
Tolerance to dogs	0.775	0.156	0.587	0.075	5.12 E−07*

Statistically significant (*P* < 0.05 ;*) difference between each paires (Bonferroni-corrected).

qu, Qualified; unq, Unqualified.

### Population genetic structure

In total, 124,675 and 154,340 SNPs in GSs (*n* = 121) and LRs (*n* = 205), respectively, were detected after over 80 and 60 K SNPs, respectively, were removed due to minor allele thresholds. These genetic data were used for all subsequent genetic analyses.

**Figure 2:**
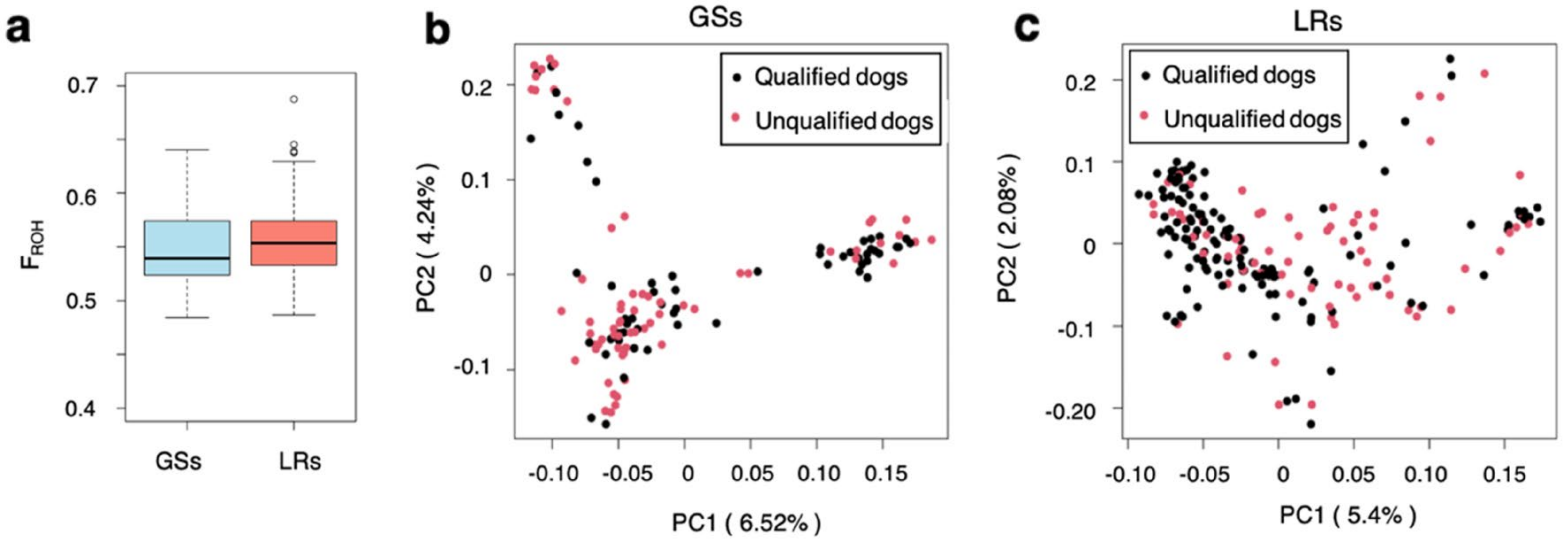
Population genetic structure in GSs and LRs. (**a**) Runs of homozygosity-based inbreeding coefficients (F_ROH_) for both breeds. Principal component analysis (PCA) in (**b**) GSs and (**c**) LRs.

The genomic inbreeding coefficients were 0.549 ± 0.003 (GSs) and 0.553 ± 0.002 (LRs) as calculated based on runs of homozygosity, which indicated no significant difference between the breeds (*t* = − 1.118, *df* = 238.06, *P* = 0.264) (Fig. 2a). Principal component analysis (PCA) revealed that a population structure was found in GSs (Fig. 2b), whereas no clear population structure was identified in LRs (Fig. 2c). No differences with statistical significance were observed between the groups of all dogs and qualified dogs.

### Genomic heritability of seven behavioral traits and qualification outcome

The heritability of the seven behavioral traits and qualification outcome was estimated based on the genomic data (Table 3). We found that heritability estimates for three traits were statistically significant: 0.84 ± 0.30 (S.E.) for ‘qualification’ and 0.82 ± 0.33 for ‘boldness’ in GSs, and 0.53 ± 0.28 for ‘tolerance to dogs’ in LRs. In GSs, ‘qualification’ and ‘boldness’ had the highest heritability among the seven behavioral traits, with a heritability estimate close to the maximum value (*i.e.*, 1), most probably owing to the low sample size and behavioral assessments at a young age. The heritability values for ‘independence’ in both breeds were extremely low and not significant.

**Table 3:** SNP heritability estimates on qualification and seven behavioral traits.

	GSs		LRs	
	*h*^*2*^_*SNP*_ (± SE)	*P* value	*h*^*2*^_*SNP*_ (± SE)	*P* value
Qualification	0.84 (0.30)	0.01	0.17 (0.32)	0.33
Activity	0.01 (0.38)	0.49	0.38 (0.31)	0.14
Boldness	0.82 (0.33)	0.04	0.14 (0.28)	0.31
Concentration	0.63 (0.38)	0.09	0.50 (0.30)	0.08
Friendliness to humans	0.49 (0.41)	0.14	0.42 (0.27)	0.06
Independence	< 0.01 (0.40)	0.50	< 0.01(0.29)	0.50
Interest in the target	0.27 (0.45)	0.31	< 0.01 (0.31)	0.50
Tolerance to dogs	0.65 (0.36)	0.07	0.53 (0.28)	0.04

GS, German Shepherd; LR, Labrador Retriever.

### GWAS of seven behavioral traits and qualification outcome

Next, GWAS was performed for seven behavioral traits and qualifications in genetically distinct GSs and LRs. The genomic inflation factors (λ) ranged from 1.020 (tolerance to dogs in LRs) to 1.131 (tolerance to dogs in GSs) (Table S1). The fact that λ was found to be larger than 1.1 only in ‘tolerance to dogs’ in GSs indicated that the other analyses were well corrected to the effect of population structure.

**Figure 3:**
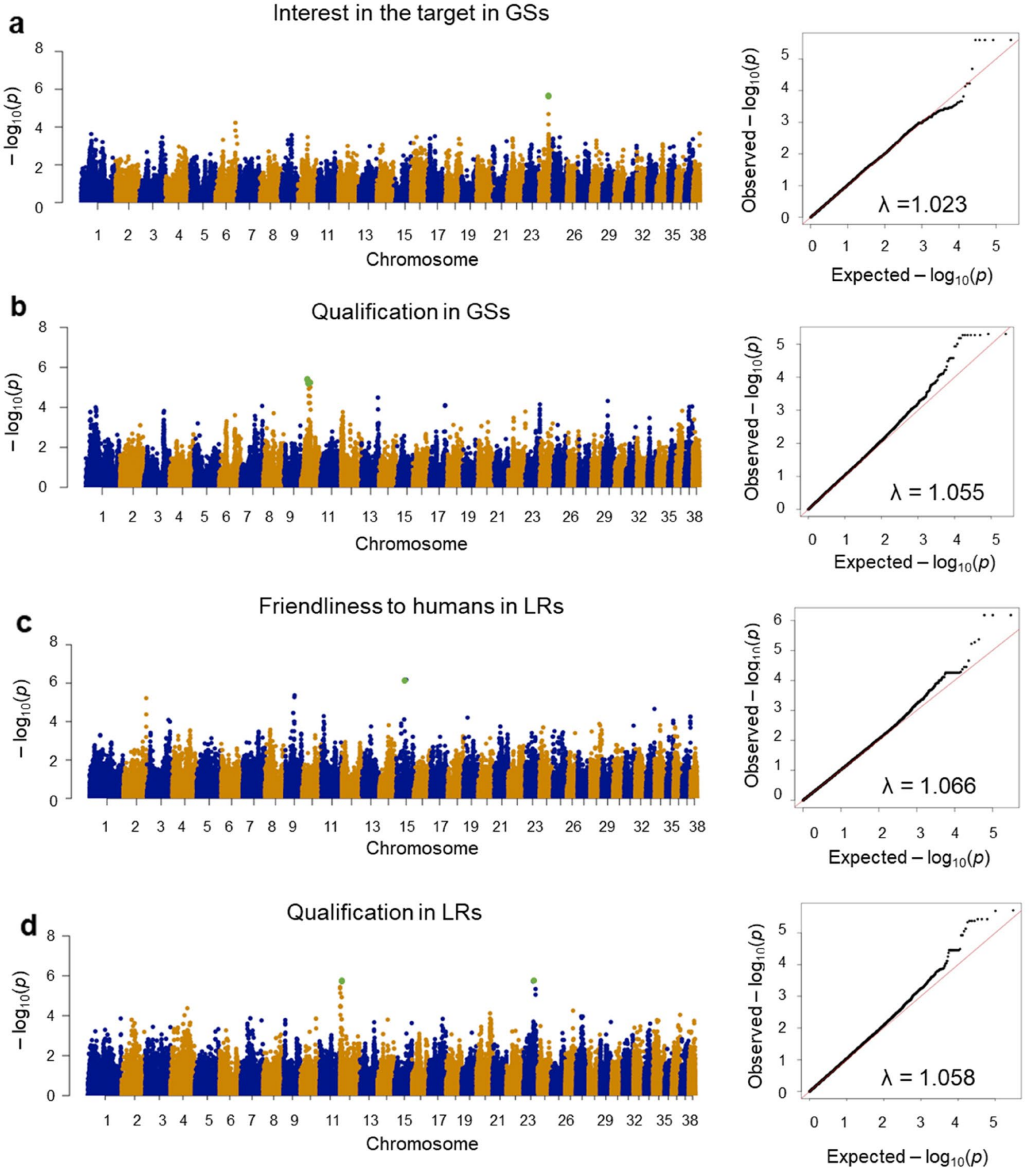
Genome-wide association study of German Shepherds (GSs) and Labrador Retrievers (LRs). Manhattan plot (left) and respective quartile-quartile plot (right) for (**a**) ‘tolerance to dogs’ in GSs, (**b**) ‘interest in the target’ in GSs, (**c**) ‘friendliness to humans’ in LRs, and (**d**) ‘qualification’ in LRs. Green dot indicates a single nucleotide polymorphism (SNP) exceeding the genome-wide significance (adjusted *P* < 0.05) and suggestive level (adjusted *P* < 0.10).

We found three genome-wide significant SNPs associated with ‘friendliness to humans’ in LRs (adjusted *P* = 0.034, Fig. 3c and Table 4). In addition, at a suggestive level, we identified five SNPs associated with ‘interest in the target’ in GSs (Fig. 3a), and ten and nine SNPs were associated with qualification in GSs and LRs, respectively (Fig. 3b, d and Table 4).

**Table 4:** Profiles of SNPs exceeding genome-wide significance and suggestive levels.

Breed	Behavioral traits	Chr	rs	Position	Allele1	Allele0	Af	Beta	SE	*P* value	Adjusted *P* value
GSs	Interest in the target	24	chr24_33618334	3,36,18,334	G	A	0.112	− 1.133	0.229	2.534 E−06	0.063
		24	BICF2G630498032	3,37,23,291	A	G	0.112	− 1.133	0.229	2.534 E−06	0.063
		24	BICF2G630498018	3,37,33,724	A	G	0.112	− 1.133	0.229	2.534 E−06	0.063
		24	BICF2G630497996	3,37,45,360	A	C	0.112	− 1.133	0.229	2.534 E−06	0.063
		24	chr24_33809914	3,38,09,914	A	G	0.112	− 1.133	0.229	2.534 E−06	0.063
	Qualification	10	BICF2G630481738	2,80,43,923	A	G	0.376	0.340	0.071	5.311 E−06	0.082
		10	BICF2G630481751	2,80,57,172	A	G	0.376	0.340	0.071	5.311E−06	0.082
		10	BICF2G630481758	2,80,62,470	A	C	0.376	0.340	0.071	5.311 E−06	0.082
		10	BICF2G630481773	2,80,89,514	A	G	0.376	0.340	0.071	5.311 E−06	0.082
		10	BICF2G630481777	2,80,96,007	A	T	0.376	0.340	0.071	5.311 E−06	0.082
		10	chr10_28136562	2,81,36,562	A	G	0.376	0.3402	0.071	5.311 E−06	0.082
		10	BICF2G630481882	2,85,23,673	A	G	0.376	0.332	0.070	6.613 E−06	0.082
		10	chr10_28523951	2,85,23,951	G	A	0.376	0.332	0.070	6.613 E−06	0.082
		10	BICF2P1224117	3,20,42,504	A	G	0.36	− 0.269	0.056	4.946 E−06	0.082
		10	BICF2P850545	3,20,60,963	C	A	0.36	− 0.269	0.056	4.946 E−06	0.082
LRs	Friendliness to humans	15	TIGRP2P198958_rs9132287	3,15,96,031	A	G	0.015	− 1.463	0.285	6.647 E−07	0.034
		15	TIGRP2P199109_rs9018263	3,21,07,172	A	G	0.015	− 1.463	0.285	6.647 E−07	0.034
		15	BICF2P472555	3,65,43,527	T	A	0.015	− 1.463	0.285	6.647 E−07	0.034
	Qualification	12	BICF2P1089244	2,13,028	G	A	0.032	0.627	0.132	4.174 E−06	0.078
		12	BICF2P1065219	2,37,954	A	G	0.032	0.627	0.132	4.174 E−06	0.078
		12	TIGRP2P155138_rs9160410	3,15,186	C	A	0.039	0.574	0.121	3.686 E−06	0.078
		12	BICF2P385657	4,07,914	A	G	0.032	0.627	0.132	4.174 E−06	0.078
		12	chr12_420732	4,20,732	G	A	0.039	0.574	0.121	3.686 E−06	0.078
		12	BICF2P843301	4,67,795	A	G	0.039	0.574	0.121	3.686 E−06	0.078
		12	BICF2S23760072	55,32,843	G	A	0.1	0.384	0.078	2.014 E−06	0.078
		23	BICF2G630371785	3,57,49,876	G	A	0.295	− 0.254	0.052	1.942 E−06	0.078
		23	chr23_44491586	4,44,91,586	G	C	0.461	− 0.215	0.046	4.609 E−06	0.078

GS, German Shepherd; LR, Labrador Retriever.

### Analysis of candidate SNPs and genes

We identified eleven regions as candidates potentially associated with the traits evaluated herein (Table S2); in particular with ‘interest in the target’ (chr24: 33,418,334–34,009,914) and ‘drug training qualification’ (chr10: 28,043,923–28,136,562, 28,523,673–28,523,951 and 32,042,504–32,107,172) in GSs, and ‘friendliness to humans’ (chr15: 31,396,031–31,796,031; 31,907,172–32,307,172; and 36,343,527–36,743,527) and ‘drug training qualification’ (chr12: 13,028–667,795 and 5,332,843–5,732,843; chr23: 3,5549,876–35,949,876 and 44,291,586–44,691,586) in LRs. A total of 63 protein-coding genes were located in these regions, but no significant term was identified upon gene ontology enrichment analysis.

Additional search using the Mouse Genomics Informatics database revealed 12 genes (Table S3) among these 61 candidates, which had phenotype annotations related to behavior/neurological systems. Considering the relatedness between behavioral traits in this study, anxiety and exploratory/social behaviors could also be valuable candidates associated with the traits studied herein. Indeed, we found that these traits were associated with seven genes, namely *Slc35c2*, *Atat1*, *Ddr1*, *Dhx16*, *Pnpla1*, *Pfn2*, and *Wwtr1*.

## Discussion

Due to limited studies on the use of drug detection dogs^19^, scientifically approved knowledge about the behavioral and genetic aspects of these animals is sparse. In the present study, we used phenotypic and genomic approaches to further explore behavior patterns and their genetic basis in relation to the successful training of drug detection dogs of two breeds. In particular, we identified nine SNPs in LRs and six genes that were potentially associated with training success of drug detection dogs. One candidate gene, the solute carrier family 35 member C2 (*Slc35c2*), is associated with decreased anxiety-related response in mice, as described in the International Mouse Phenotyping Consortium database (https://www.mousephenotype.org/). In addition, mice with mutated profilin 2 (*Pfn2*) were reported to exhibit higher novelty-seeking behavior than wild-type animals^20^. Although these studies were performed in mice, their findings support our results in dogs. Further studies are required to obtain better knowledge of genetic factors underpinning behavioral features and to extend our understanding of the influence of these factors on individual differences in the responsiveness of drug detection dogs to intensive training.

Behavioral differences between dog breeds are widely known and well-documented^21^. Our behavioral analysis contributed to this body of knowledge by elucidating breed differences with respect to ‘friendliness to humans’ and ‘tolerance to dogs’ traits between GSs and LRs. In addition, we found moderate genomic heritability related to ‘friendliness to humans’ in both breeds. These findings indicate that behavioral traits may be influenced by genetic factors and that the genetic basis may be similar among dog breeds.

GWAS has successfully identified SNPs associated or potentially associated with various behavioral traits in dogs^12,13^. Compared with a previous study, the sample size of our study was smaller (*n* < 500 in both breeds). There are two factors that can possibly contribute to successful identification of candidate regions: (1) phenotyping and (2) standardized environment or the use of covariates. The first factor is related to the method of phenotyping. The C-BARQ, which is widely utilized to collect behavioral GWAS data in dogs^12,13,22^, is usually used only once for behavioral evaluation, which may potentially reflect behavioral traits of the dog at the time of the evaluation (although the C-BARQ has been standardized on the basis of reliability and validity^23^). Here, we used two types of behavioral phenotyping of dogs, namely estimation of seven behavioral traits during the initial training and the final training success (qualified/unqualified). Similarly, only one person (usually the owner) answers the questions in the C-BARQ, whereas in our approach, the final training success was judged by several dog experts from the training facility. We attempted to detect genetic regions associated with behavior by using multiple phenotypic assessments, but improving the reliability and validity of behavioral assessments is a challenge for future experiments (see below). Although further studies are needed to compare the results of standard C-BARQ and our approach, phenotyping as performed herein should be acceptable as compared with the standard method thanks to the aforementioned two reasons.

As environmental factors generally affect dog behavior^24^, the animals herein evaluated shared the same housing facility from 1 year of age and the same trainers, which resulted in lower environmental variance than that in common households, which were the setting of the previous reports. Although one of the limitations involved in the uncontrollable environmental factors of socialization since the day of birth to about 1 year of age, GWAS models also included the kennel information, so environmental factors were therefore at least partially controlled for, and standardized environmental factors thereafter could be controlled at least partially. Taken together, highly consistent data for behavioral traits and low environmental variances should provide a good basis for obtaining better results. However, increasing the number of phenotyped dogs in future studies could improve the detection power of genetic variants influencing behavior relevant for the performance of drug detection dogs.

Estimating heritability is one of the primary methods used to evaluate genetic effects on phenotypic traits. Traditionally, heritability estimates are based on pedigree^25^; however, significant technological improvements have recently accelerated heritability estimation based on genomic data (genomic heritability)^11,12^. Both pedigree- and genomic-based heritability estimates are influenced by the quality of phenotyping (scoring of traits) and environmental factors. We have identified higher heritability estimates with statistical significance in behavioral traits than previously reported (0.00–0.16^13^ and 0.00–0.23^12^). This difference may likely relate to the way the traits were evaluated, standardized and controlled environmental factors used in the models, including covariates as discussed above, as well as genetic relationships within the dog population, as reported in humans^26^. Another possible factor associated with high heritability is the age of the study population. A behavioral genetic study in mice reported that heritability was higher when behavioral assessments were performed in younger, 6-week-old, sexually mature animals than in older, 8-week-old mice^27^. Comparative studies with humans suggest that the aging process in mice can be faster than that in dogs^28,29^, and the age of dogs in our study (i.e., about 1 year of age) corresponds to early stages of sexual maturity. Considering these reports, the younger age of the studied dogs than previous studies possibly contributes to estimating higher heritability.

Taken together, our results contribute to a better understanding of the genetic effects on behavioral traits related to the successful training of drug detection dogs, which may in turn support improved breeding and training protocols for working dogs. Further analyses using more comprehensive data sets (*i.e., n* > 500) with high-quality behavioral phenotyping are needed to validate our findings. For instance, future studies should assess dogs’ behavior using methods that are based on measurements of actual behavioral parameters rather than questionnaire surveys and qualitative evaluations. A leading candidate for behavior tests is the Dog Mentality Assessment (DMA), which has been developed to assess the suitability of working dogs such as police and service dogs. Although no GWAS studies have been applied to the DMA data yet, the DMA has been standardized with regards to its validity and reliability in large samples of dogs^30,31^^.^ In fact, dogs with higher DMA 'boldness' scores are more likely to be certified as working dogs than those with lower scores^16^. Until now, the training and evaluation of working dogs, including scent detection dogs, have been developed and refined through unique methodologies at training facilities in various regions. However, it is expected that dog behavior evaluation systems will be shared worldwide, and more effective standards of dog behavior will be developed in the future. The present study is considered the first step for further development of the scent detection dog training system in Japan.

## Methods

### Animals and training protocol

All dogs used in this study were either purebred German Shepherds (GSs, *n* = 127) or purebred Labrador Retrievers (LRs, *n* = 497), which are the most common dog breeds used as drug detection dogs by Japan Customs.

The dogs were trained for drug detection at the Canine Training Center, Tokyo Customs, between 2002 and 2019. They were provided by dog breeders and removed from the general dog population (and not from specialized kennels) at approximately 1 year of age. The candidate dogs did not receive any specific training for scent detection until then. At the training center, the dogs were kept in kennels, and the animal management staff provided daily care in accordance with the relevant regulations in Japan. We could not obtain the precise pedigree data for confidentiality reasons by Tokyo Customs (*i.e.,* the information about the dogs could potentially affect the future and training in the customs). Instead, we obtained data about the kennel (i.e., breeder) where the dog was born and used it as a covariate in heritability estimation (see Genomic heritability estimation).

Dogs were trained to find a range of different substances (*e.g.,* marijuana, hashish, cocaine, heroin, and methamphetamine) that are prohibited for import, export, or possession in Japan. The training method was based on positive reinforcement with social rewards; food rewards were not used. Canine trainers played tug-of-war with the dogs using a rolled towel with a target scent (*i.e.,* reinforcer or “target”), so that the dogs were motivated to search for an object with the scent. The training period lasted for approximately 4 months and was divided into three phases: familiarization, basic training, and advanced training. The familiarization phase aimed at habituating the dogs to the training methods and novel environment of the training facility. Basic and advanced training phases aimed at training the dogs to detect drugs with strong scent (*e.g.,* marijuana and hashish) and soft scent (*e.g.,* cocaine, heroin, and methamphetamine), respectively. The success/failure of dog training for scent work was determined by a double-blind detection test for each dog-handler pair at the end of each training phase. In the detection test, the dog-handler pair searched a target scent in different situations (*e.g.,* baggage on a belt conveyor, travelers’ carry-on items, packages at the postal office, and import cargo). Based on the performance of the dog and its handler in a test situation, a panel of supervisors (*i.e.,* not the dog’s handler) at the training facility discussed and determined which dogs were successful for scent detection work. Candidate dogs had to exceed the standard score in all test situations to advance to the next training phase. The protocol of dogs' performance evaluation has been standardized in the training facility, but details of the scoring method were not disclosed for confidentiality reasons. Only dogs that passed all three training phases moved to on-site trials to test their behavioral flexibility in the context of working situations. Dogs that succeeded during the on-site trials were employed for drug detection in the field (*e.g.,* airport or port) and were categorized as ‘qualified,’ whereas those that did not were categorized as ‘unqualified.’ Thus, the training success or failure of dogs in this study was a complex trait formed by training history based on the interaction between handler and dog, although it was partially determined using controlled tests.

Finally, 121 GSs and 205 LRs were used for genomic analysis, corresponding to sampling proportion of 64/121 (52.9%) in GSs and 80/205 (39.0%) in LRs for qualified dogs, respectively. Previous studies have suggested that the proportion of successful training of working dogs reaches 30–50%^17,32^, which is consistent with the sampling proportion per breed of the present study. It must be noted that these sampling proportion of dogs for possible detailed genetic analysis are not identical to the proportion of qualification for field work. Japan Customs reported that the proportion of qualification is approximately 30% for both breeds. The outline of the training protocol has been described in further detail in previous studies^4,5^. This study was approved by the Ethics Committee of the Wildlife Research Center, Kyoto University (WRC 2010EC001) and performed in accordance with the relevant guidelines and regulations. This study used a non-invasive method based on behavioral observation, plus blood sampling for obtaining material for molecular genetic analysis (SNP genotyping). All methods using dogs in this study were reported in accordance with the ARRIVE guidelines (https://arriveguidelines.org).

### Assessment of behavioral traits

To quantify the behavioral characteristics of the dogs, we asked the training staff to score seven behavioral traits that could be related to the success of detection training. Candidate dogs were assessed only once after 2 weeks of the familiarization phase of training (see Animals and training protocol). This allowed us to evaluate the relatively ‘naive’ behavioral characteristics of a candidate dog at a less advanced stage of scent detection training. For each dog, activity (*i.e.,* the amount of general activity typically observed), boldness (*i.e.,* low fearfulness displayed toward unfamiliar environments), concentration (*i.e.,* attention span during training), friendliness to humans (*i.e.,* willingness to interact with humans), independence (*i.e.,* self-confidence, willingness to work without relying on humans), interest in the target (*i.e.,* degree of interest in a reinforcer (rolled towel), and tolerance to dogs (*i.e.,* low aggressiveness towards other dogs) were evaluated (Table 1). We asked two training managers with at least 10 years of experience to assess the behavioral traits of each dog. Throughout the study period, either one of the two training managers (*i.e.*, managers A or B) working at the training facility at that time rated the extent to which a behavioral trait was applicable to each dog using a 5-point scale with a score of 5 indicating ‘very high’. Seven behavioral traits examined in this study have been used to assess dogs’ performance in the facility. The dogs scoring higher in each trait were considered as more suitable for drug detection. Actually, the previous study demonstrated that four of these traits (activity, boldness, concentration, and interest in target) were significantly associated with successful dog training^5^. Generally, the use of ‘subjective’ ratings using questionnaires has the advantage of extracting individual characteristics independent of a specific time or situation^33^ (although assessments of dogs' performance using methods more reliant on intrinsic behavior have also been developed^16,30,31,34^). Although objective evaluation of behavior was not possible in this study, an equipment and protocol for recording behavior during odor search are currently under development. The detailed procedure of behavioral assessment has been described in previous studies^4,5^.

Behavioral differences based on sex, qualification status, and breed were determined using the Wilcoxon rank-sum test implemented in R software (version 3.6)^35^. Multiple comparisons (seven traits based on sex, qualification, and breeds) were conducted and the Bonferroni correction was applied. The significance level of the corrected *P-*value was 0.0014 (0.05/35).

### Genotyping

Genomic DNA of all dogs was extracted from blood samples, which were obtained using a DNeasy Blood and Tissue Kit (Qiagen, Hilden, Germany), according to the manufacturer’s instructions. The Canine 230 K Consortium BeadChip Array (Illumina, San Diego, CA, USA) was used for genome-wide SNP genotyping following the standard protocols provided by the manufacturer. Prior to genomic analyses, quality control of the samples and markers was performed by breed using PLINK v1.90^36^ and v2.00a2LM with the following settings: missingness per dog < 0.05; minor allele frequency > 0.01; missingness per marker < 0.05; sex-check by correspondence of genetic sex to that indicated in the interview sheet; removal of genetically identical dogs based on kinship values exceeding 0.354; exclusion of mitochondrial SNPs, SNPs located on the sex chromosomes, unlocalized SNPs.

### Analysis of population genetic structure

We determined the genomic inbreeding coefficients and performed PCA to evaluate the genetic structure of the original population. Genomic inbreeding coefficient was calculated based on runs of homozygosity (F_ROH_) using PLINK v1.90 and detectRUNS v.0.9.6 R package (settings: maxOppRun = 0, maxMissRun = 0, minSNP = 2, minLengthBps = 100, and maxGap = 500,000), while applying the methods of sliding windows^37^ and consecutive runs^38^. Differences in F_ROH_ between breeds were tested using the Welch’s two-sample *t*-test implemented in R. Statistical significance was set at 0.05. PCA was performed using the *–pca* option implemented in PLINK v1.90.

### Genomic heritability estimation

Genomic heritability of each behavioral trait and qualification status as drug detection dogs was estimated using the Genome-wide Complex Trait Analysis (GCTA) software v1.91.7beta^39^. The log-likelihood ratio test was used to estimate genetic variance, residual variance, phenotypic variance, and standard errors. Genomic heritability was estimated based on genome-wide SNP data as a ratio of genetic variance to phenotypic variance. SNP heritability (*h*^2^_SNP_) was tested using the Genomic Restricted Maximum Likelihood method implemented in GCTA software. The kennel of each dog and training period (reflecting the effect of trainers and judges) were used as covariates.

### GWAS

A linear mixed model was fitted using GEMMA software^40^. We tested the associations between SNP genotypes and behavioral scores for the seven behavioral traits (1–5) or binary for qualification (qualified or unqualified). To handle the effect of population structure on the GWAS, we used a centered relatedness matrix option (*− gk 1*) in GEMMA as a random effect. The kennel of each dog and training period (the effect of trainers and judges) were used as covariates. *P*-values were calculated using the Wald test. The genomic inflation factor (λ) was calculated using the R package GenABEL v.1.8. to estimate the effect of the population genetic structure on GWAS results. The λ values were estimated using a regression model. To address the multiple testing problem, the proportion of false positive results was calculated using the *p.adjust* function in R software (v.3.6). The genome-wide significance and suggestive level thresholds for adjusted *P*-values were set at 0.05 and 0.10, respectively.

### Analysis of candidate SNPs and genes

A subsequent analysis based on linkage disequilibrium was performed to identify potential genes related to each trait. We denoted the candidate loci associated with traits as follows. Candidate SNPs exceeding significance and suggestive level thresholds were subjected to uncover the candidate genes. All gene datasets were obtained from the Ensembl genome browser (release 104, CanFam 3.1). We denoted the length between adjacent candidate SNPs, including significant and suggestive, as within 200 kb. When the SNP did not share the high-LD SNPs, then the region located ± 200 kb of the SNP was denoted as the same locus for target^14^. Bedtools v2.27.1 was used to extract the Ensembl gene IDs from the gene feature format file downloaded from the Ensembl genome browser.

To determine the functional classes of genes associated with the analyzed traits, we used the gene ontology enrichment analysis powered by PANTHER^41^. The target genes were selected using the above linkage disequilibrium-based analysis. PANTHER Overrepresentation Test (Released 20,210,224) was used. ‘Biological process’ was used for annotated analysis with PANTHER 16.0. for domestic dog datasets (*Canis lupus familiaris*) as the analysis type. Ensembl gene IDs were used for all annotation data.

As in vivo evidence in mice facilitates the search of genes associated with traits, we used the Mouse Genomics Informatics database (v 6.17) to investigate the relationships between genes and phenotypes (http://www.informatics.jax.org/); phenotype annotations related to ‘behavior/neurological’ category were targeted to identify the relationship.

## Supplementary Information

Supplementary Tables.

## Data Availability

All SNP and behavioral data were deposited in the Dryad database (https://datadryad.org/stash/share/vVhbcaRDt98fHq1Avzkg0sVRdtlQ7jf-zd5FNnq318Y).
